# Indigenous Peoples' Data During COVID-19: From External to Internal

**DOI:** 10.3389/fsoc.2021.617895

**Published:** 2021-03-29

**Authors:** Stephanie Russo Carroll, Randall Akee, Pyrou Chung, Donna Cormack, Tahu Kukutai, Raymond Lovett, Michele Suina, Robyn K. Rowe

**Affiliations:** ^1^College of Public Health, University of Arizona, Tucson, AZ, United States; ^2^Native Nations Institute, University of Arizona, Tucson, AZ, United States; ^3^Department of American Indian Studies and Public Policy, University of California, Los Angeles, Los Angeles, CA, United States; ^4^Open Development Initiative, East West Management Institute, New York, NY, United States; ^5^Te Kupenga Hauora Māori, Faculty of Medical and Health Sciences, University of Auckland, Auckland, New Zealand; ^6^National Institute of Demographic and Economic Analysis, The University of Waikato, Hamilton, New Zealand; ^7^Research School of Population Health, Australian National University, Canberra, ACT, Australia; ^8^Albuquerque Area Indian Health Board–Albuquerque Area Southwest Tribal Epidemiology Center, Albuquerque, NM, United States; ^9^School of Rural and Northern Health, Laurentian University, Greater Sudbury, ON, Canada

**Keywords:** Indigenous Peoples, data sovereignty, data governance, data sharing, public health

## Abstract

Global disease trackers quantifying the size, spread, and distribution of COVID-19 illustrate the power of data during the pandemic. Data are required for decision-making, planning, mitigation, surveillance, and monitoring the equity of responses. There are dual concerns about the availability and suppression of COVID-19 data; due to historic and ongoing racism and exclusion, publicly available data can be both beneficial and harmful. Systemic policies related to genocide and racism, and historic and ongoing marginalization, have led to limitations in quality, quantity, access, and use of Indigenous Peoples' COVID-19 data. Governments, non-profits, researchers, and other institutions must collaborate with Indigenous Peoples *on their own terms* to improve access to and use of data for effective public health responses to COVID-19.

## Introduction

Governments have used real-time data and disease trackers quantifying the size, spread, and distribution of the novel coronavirus SARS-CoV-2 (COVID-19) that emerged in 2019 to inform and influence decision and policy making. Indigenous Peoples have been disproportionately affected by COVID-19, whether through infection, fatality, economic losses, or changes to social interactions. While Indigenous Peoples need timely, relevant, high-quality data to inform their own pandemic response, the collection and use of such data are not without risk (Carroll et al., 2020c; Curtice and Choo, 2020; del Pino and Camacho, 2020; Nagle, 2020; Paulin, 2020; RNZ, 2020; Tahir and Cancryn, 2020). In recent months, concerns have been raised around data harms, group privacy, consent, racist surveillance, algorithmic profiling, and more (Carroll et al., 2020c; Furlow, 2020a,b; Paulin, 2020; Timothy, 2020; United Nations, Department of Economic and Social Affairs, 2020; World Health Organization, 2020).

This paper explores the particular issues that COVID-19 has highlighted for Indigenous Peoples focusing on data for governance. Drawing on current global examples, we underscore the inclusion of Indigenous Peoples in COVID-19 activities as the basis of data-related policy recommendations to increase the use of timely, relevant data for decision-making while reducing risk and harms.

## Indigenous Peoples: Data, Sovereignty, and Governance

Over 370 million Indigenous persons belong to more than 5,000 diverse cultures and inhabit over 90 countries worldwide (United Nations, 2009). However, this is a dated and gross underestimation, particularly for certain countries (e.g., low income) and regions; the most recent assessment from Asia alone estimates over 411 million Indigenous persons living in those countries (Asia Indigenous Peoples Pact, 2019). Indigenous Peoples as political collectives with inherent sovereignty share continuity with their pre-colonial societies (Martinez Cobo, 1982). Through their own social, political, and economic systems, Indigenous Peoples preserve, develop, and transmit their cultures, knowledge's, and relationships with their territories and resources to future generations (Martinez Cobo, 1982). In this paper we use Indigenous Peoples, nations, and communities together to denote the variety of ways in which Indigenous Peoples organize and refer to themselves. When referring to a specific Indigenous Peoples, we use the preferred terminology of those peoples (e.g., Pueblo of, First Nation, Māori).

Indigenous data are information or knowledge in any format that impact the lives of Indigenous Peoples collectively and individually, including data about lands and resources; information about individuals; and collective cultural and traditional knowledges (Maiam nayri Wingara, 2018; Te Mana Raraunga, 2018; Carroll et al., 2019, 2020a; Rainie et al., 2019). In the COVID-19 context, Indigenous data comprise information about COVID-19 testing (including community level measures such as municipal waste water), cases, hospitalizations, health service access, deaths, and comorbidities (Research Data Alliance COVID-19 Indigenous Data Working Group, 2020). Indigenous data also encompass related Indigenous Knowledges about COVID-19 and information on the socioeconomic and environmental correlates and impacts of COVID-19 (Research Data Alliance COVID-19 Indigenous Data Working Group, 2020). Data include information and metrics (i) for Indigenous Peoples as defined by geographic jurisdiction, (ii) for community members, and (iii) that include Indigenous nation-affiliation or Indigenous identifiers or affiliation (e.g., nation, tribe, ethnicity) within nation-state and local data systems. These are all data about Indigenous Peoples, lands, and resources, regardless of where individuals reside or where the data are held.

Epistemicide through settler colonial practices that have suppressed and co-opted Indigenous knowledge systems has created a state of data dependency (Carroll et al., 2019). Inconsistent, inaccurate, and irrelevant data; lack of Indigenous control and ownership of data; negative experiences with untrustworthy, exploitative research and policy practices; lack of investment in Indigenous Peoples' data infrastructures and capacity; and deficit focused data use and application mark and perpetuate data dependency (Kukutai and Taylor, 2016; Rodriguez-Lonebear, 2016; Walter, 2016; Rainie et al., 2017b). Within epidemiology specifically, these issues are also apparent for Indigenous Peoples in relation to data on Indigenous health and well-being (Anderson et al., 2016; Paradies, 2016; Prussing, 2019; Paine et al., 2021). As a result, many Indigenous nations rely on other governments, organizations, and institutions to provide both the data about their communities and the expertise to use and apply those data (Kukutai and Taylor, 2016; Rodriguez-Lonebear, 2016; Snipp, 2016; Carroll et al., 2019). Furthermore, outside data professionals often become the experts to which both Indigenous nations and other entities refer to for information and analysis using Indigenous Peoples' data (Smith, 2012; Walter and Andersen, 2013; Kukutai and Taylor, 2016). Indigenous Data Sovereignty serves to counter these actions and this narrative, recognizing and revitalizing Indigenous Knowledges to guide data practices.

Indigenous Data Sovereignty draws upon the United Nations Declaration on the Rights of Indigenous Peoples which reaffirms the rights of Indigenous Peoples to govern the collection, application, re/use, and stewardship of their data (United Nations, 2007, 2018, 2019; Davis, 2016; Kukutai and Taylor, 2016; Snipp, 2016; Rainie et al., 2017a). Substantial variations in nation states' recognition of sovereignty across the globe differentially affect (1) the existence, availability, and access to COVID-19 related data for Indigenous Peoples and (2) Indigenous Peoples' assertions of self-determination and data governance. In contrast to Indigenous Peoples in Aotearoa New Zealand, Australia, Canada, and the United States (US), in low- and middle-income countries Indigenous Peoples (1) have even less access to data and information, (2) represent a large proportion of Indigenous Peoples worldwide, and (3) suffer increased persecution during the pandemic crisis (del Pino and Camacho, 2020; United Nations, Department of Economic and Social Affairs, 2020; World Health Organization, 2020). Currently, the only COVID-19 data for developing countries is available through the United Nations Humanitarian Data Exchange^1^. Unfortunately these data are reported at nation-state level due to sensitivities and are not disaggregated by race or ethnicity. Thus, the majority of the examples presented here address COVID-19 data situations in Aotearoa New Zealand, Australia, Canada, and the US. Yet similarities in COVID-19 data challenges for Indigenous Peoples exist internationally (del Pino and Camacho, 2020; United Nations, Department of Economic and Social Affairs, 2020; World Health Organization, 2020).

The CARE Principles for Indigenous Data Governance (Collective benefit, Authority to control, Responsibility, Ethics) are propelling international discussions around Indigenous Peoples' data during a global pandemic and beyond (Research Data Alliance International Indigenous Data Sovereignty Interest Group, 2019; Carroll et al., 2020a,b; Research Data Alliance COVID-19 Indigenous Data Working Group, 2020). Using the CARE Principles as a framework, the Research Data Alliance (RDA) COVID-19 Working Group set forth guidelines that underscore the need to engage Indigenous Peoples across COVID-19 data lifecycles and ecosystems (Research Data Alliance COVID-19 Indigenous Data Working Group, 2020). These guidelines demand investments in Indigenous community governance and control of data, while also making visible the information access and quality challenges that restrict the flow and use of COVID-19 data for public health response. Additionally, Indigenous Peoples' have rights to self-determine COVID-19 responses and to participate in broader decision-making (United Nations, Department of Economic and Social Affairs, 2020; World Health Organization, 2020).

## Data for Governance

Governments, institutions, corporations, health care systems, and individuals require relevant and timely data for decision-making with respect to COVID-19 and future pandemic planning, mitigation, and surveillance. Indigenous Peoples and nations need these data for governing, determining policy, and evaluating program performance (Rodriguez-Lonebear, 2016; Smith, 2016; Snipp, 2016; Rainie et al., 2017b). These data also provide a lens to assess the impact of COVID-19 emergency response efforts at the national, regional, and local levels. Systemic policies related to genocide and racism, historic and ongoing marginalization, have led to lack of timely, accessible and representative COVID-19 data for Indigenous Peoples (Rodriguez-Lonebear, 2016; Walter, 2018; Carroll et al., 2019). There is a long history of lack of epidemiologic and other data for Indigenous Peoples (Anderson et al., 2006, 2016; Gracey and King, 2009; King et al., 2009; Axelsson et al., 2016; Paradies, 2016; Agyepong et al., 2017). To complicate the lack of data, existing laws and relationships are often ignored; for instance in the US, Indigenous nations are public health authorities with the same rights and responsibilities as state and local governments, yet many state governments refuse to share COVID-19 data with them (Tahir and Cancryn, 2020).

Many Indigenous Peoples across the globe lack access to data disaggregated by Indigenous affiliation or identification (e.g., nation, tribe, ethnicity). In the US, disaggregated data were not available for COVID-19 infection rates for Indigenous Peoples at the start of the pandemic (Nagle, 2020). This same scenario still exists in some US states and across the globe (Curtice and Choo, 2020; Hatcher et al., 2020; United Nations, Department of Economic and Social Affairs, 2020; World Health Organization, 2020). Misclassification or lack of classification on death certificates also leads to unavailable or underreported COVID-19 mortality data (Carroll et al., 2020c; Peeler, 2020). As a result, Indigenous Peoples lack the data to track the size, spread, and distribution of cases and fatalities for Indigenous nations and populations (both within and outside of Indigenous communities) for prevention, surveillance, mitigation, and evaluation purposes.

However, in the absence of Indigenous Peoples' participation in decision-making and access to data held by others during the COVID-19 pandemic, risks of data weaponization, stigmatization, and racialization rise (Carroll et al., 2020c). In the US, a COVID-19 hospital policy racially profiled pregnant Native American women using zip code level COVID-19 data made public by the State of New Mexico on an online dashboard (Furlow, 2020a). In the state where the hospital is located, COVID-19 case data are made public on an online dashboard by the state government. COVID-19 case data on tribal lands are included on this dashboard without tribal permission. A federal investigation found that the hospital singled out Native American patients with reservation zip codes by requiring them to undergo COVID-19 testing even though they did not necessarily have a higher risk for exposure to the virus to stop the spread of the disease (Furlow, 2020b). The hospital also failed to provide explicit options for either refusing or requesting the testing. Furthermore, some of the affected mothers and newborn babies were separated during an important period of postpartum bonding while awaiting test results. For Native Americans, the hospital's discriminatory and unethical policy is even more problematic because it is reminiscent of US Federal Indian Policy that allowed for children to be removed from their homes and separated from their families and communities during the boarding school era.

In Australia, recommendations regarding protecting “vulnerable people” from COVID-19 included that “People aged 70 and over should stay at home and self-isolate for their own protection to the maximum extent practicable,” and that “These arrangements should also apply to those with chronic illness over 60 and Indigenous persons over the age of 50” (Rev, 2020). While this advice appears precautionary, it is insulting for Indigenous Peoples and there is no information available to indicate how the advice was formed or what data were used to inform the advice. As a result, the advice for Indigenous people was later amended to those who are 50 years *with a chronic health condition*(s) to reflect a more generic and correct broader statement for those with chronic health conditions (Australian Government Department of Health, 2020). Indigenous Peoples require access to accurate data to understand the extent of risk and evaluate policy statements about risks.

In many places we have seen the continued underfunding of Indigenous public health during the pandemic, which further limits the access and use of data for the distribution of resources and investments in infrastructure and immediate needs. Additionally, even when funds are provided, often they come with strings such as excess regulation and micro-management and/or fear from Indigenous Peoples to assert their own governance systems and Indigenous knowledge's to curb the spread of SARS-CoV-2 (Carroll et al., 2020b). When the COVID-19 pandemic hit, an $82 billion emergency response package was announced by the Canadian federal government for the country (Harris, 2020). Within that, two separate funds totaling $305 million were announced by the federal government to address the specific and immediate pandemic needs of First Nations, Inuit, and Métis peoples (Government of Canada, 2020). This amount is less than proportionate to investments in the general Canadian population (Yellowhead Institute, 2020). This lack of sufficient resource allocation limits Indigenous efforts to track and mitigate the spread of the virus. Improved funding could lead to better community data infrastructure, greater capacity development, and ultimately decrease the potential for negative outcomes relating to COVID-19.

The COVID-19 pandemic has been used to apply further restrictions to already marginalized groups. In Myanmar there has been a mobile internet shutdown in some areas, movement restrictions implemented, and a blocking of aid that have a significant potential to cause a major COVID-19 outbreak in camps (Human Rights Watch, 2020). The continued invisibility of Indigenous Peoples in COVID-19 data effectively erases their existence, paving the path for continued harms. Ameliorating these challenges requires actions to support Indigenous Peoples' access to and use of data.

## Actionable Recommendations

This section outlines tangible data-related policy recommendations for governments, non-profits, researchers, and other institutions that emerge from the del Pino and Camacho (2020); Research Data Alliance COVID-19 Indigenous Data Working Group (2020); United Nations, Department of Economic and Social Affairs (2020); World Health Organization (2020), scholars (Anderson et al., 2006, 2016; Gracey and King, 2009; King et al., 2009; Axelsson et al., 2016; Paradies, 2016; Agyepong et al., 2017), and Indigenous Peoples and allies worldwide.

### Invest in Indigenous Community-Controlled Data Infrastructures and Technology to Support Community Capacity, Response, and Resilience

Data collection in, and repatriation to, Indigenous communities is required to ensure communities and nations can make decisions affecting them. Data creation, storage, and use by Indigenous Peoples necessitates investments in community-controlled data infrastructure and technology. In Australia, there are early indications of establishing regional data infrastructure so Indigenous nations can use data for development (National Agreement on Closing the Gap, 2020). One policy proposal to assist in the current COVID-19 environment for Indigenous communities to respond to COVID-19 is to invest in technological solutions such as a syndromic surveillance system for Indigenous community-controlled/based data systems where there are options to do so, and appropriate Indigenous data governance can be applied. Syndromic surveillance is where automated generation of statistical alerts through monitoring of disease indicators can occur in real time or near real-time to detect potential outbreaks of disease earlier than would otherwise be possible with traditional public health methods (Henning, 2004).

Even in low resource, rural environments options exist to support community-data infrastructure. In Brazil, the Coordination of the Indigenous Organizations of the Brazilian Amazon and the Amazon Environmental Research Institute collaborated on a mobile application (app); the “COVID-19 Indigenous Alert” app assists Indigenous communities in monitoring the spread of the pandemic on their lands and informing mitigation efforts (IAPM, 2020). The Kuikuro Indigenous Association of Upper Xingu in Brazil further customized their response by enhancing already existing data infrastructure, collaboration, and governance activities for territories and sacred sites to serve as a COVID-19 monitoring and tracing app (Contreras, 2020; Dias, 2020). Nested within other mitigation techniques, the app has proven useful in controlling outbreaks.

### Involve Indigenous Peoples' Leaders, Activists, and Scholars in the Mainstream Science/Data/Policy Nexus Decision-Making Processes

Essential to good governance is good decision making. For populations where decisions need to be made, involving people and communities from those populations in decision making is core to governance (Cornell and Kalt, 2000; Jorgensen, 2007). During the pandemic, Australia implemented a policy to involve Aboriginal and Torres Strait Islander community organizations and experts in a Taskforce for planning and decision making (Australian Government, 2020). The Taskforce is co-chaired by the peak national group representing Aboriginal Community Controlled Health Organizations and the Australian Government Department of Health. The group convened in March 2020 to create a COVID-19 national management plan made up of several activities including establishment of community controlled respiratory clinics; point of care testing; development of online training COVID-19 infection control training; advice on community preparedness and communications.

In Aotearoa New Zealand, Māori rights to self-determination and inclusion in nation-state governance, which includes data governance, is grounded in the nation's founding constitutional document the 1840 Te Tiriti of Waitangi (Treaty of Waitangi) (Ruru, 2016). The last decade has seen growing pressure on public sector institutions to embed and implement policies and practices that give effect to Te Tiriti, particularly in the health sector (Waitangi Tribunal, 2019). Early on in the pandemic, Māori openly challenged the government's failure to meaningfully include them in pandemic response decision-making (Kukutai et al., 2020). Tribal and community leaders, some of whom set up their own community-controlled checkpoints, questioned the government's exclusive authority to make decisions in the best interests of Māori. The national Māori pandemic group, Te Ropu Whakakaupapa Urutā, called for a “by Maori, for Maori, about Maori” response strategy (Te Ropu Whakakaupapa Urutā, 2020). Key to the strategy is the critical need for reliable, routinely-available and ethnicity disaggregated data and real-time monitoring to inform Māori sovereign decisions.

### Institute Data Access and Sharing Protocols Between Indigenous Peoples and Other Governments and Data Holders

In Canada, the First Nations Information Governance Centre's Principles of OCAP® (Ownership, Control, Access, and Possession) have contributed to the development of research policy and practice for the governance of First Nations' information (First Nations Information Governance Centre, 2020). For instance, the Government of Canada's Tri-Council Policy statement that guides ethical research includes specification in Chapter 9 for Research Involving the First Nations, Inuit and Métis Peoples' in Canada (Canadian Institutes of Health Research et al., 2018). Chapter 9 acknowledges and respects the diversity of Indigenous people's lives and experiences throughout the research process (Canadian Institutes of Health Research et al., 2018). Within these frameworks, the administrative health data holders, known as Institute for Clinical Evaluative Sciences (ICES), and the Chiefs of Ontario (COO), the coordinating body for the 133 recognized First Nations communities in the province of Ontario, entered into a Data Governance Agreement. This agreement honors the First Nations' Principles of OCAP® and enables ICES, as the provincial data custodian, to carry out health-related analyses at the request of COO and the First Nations communities that COO supports and advocates on behalf of Pyper et al. (2018). During COVID-19, the existing Agreement allowed First Nations' communities and leadership to access timely and reliable information to respond quickly. Challenges persist, as funding and support limitations restrict improvements in First Nations' data availability, infrastructure and capacity (Trevethan, 2019).

Tribes in the US have had varied success in accessing their COVID-19 data held by the federal and state governments (Carroll et al., 2020c; Nagle, 2020; Tahir and Cancryn, 2020). Some states have denied tribal sovereign rights to control sharing of their COVID-19 data (Carroll et al., 2020c). In recognition of Indigenous Data Sovereignty, the State of Arizona withheld tribal zip code data until tribal data sharing permissions were obtained to publicly release tribal data. Data sharing involves both the release of tribal information as well as the sharing of tribal information that other governments hold, such as COVID-19 related data, with tribes. A number of states and the federal government have denied tribal rights to access data for decision-making (Tahir and Cancryn, 2020). There is a recognized need for other governments and organizations to establish data sharing agreements and mechanisms with Indigenous Peoples (Research Data Alliance COVID-19 Indigenous Data Working Group, 2020; United Nations, Department of Economic and Social Affairs, 2020; Urban Indian Health Institute, 2020; World Health Organization, 2020). Indigenous nations' own codes and policies can be instituted to bolster this response, such as data sharing requirements and relationships as part of tribal public health codes in the US (Hiraldo et al., 2021).

### Require Collection (and Validation) of Indigenous Identifiers or Affiliation (e.g., Nation, Tribe, Ethnicity)

Decision making relies on accurate reliable information. Data used for Indigenous nation and community decision making relies on collection of Indigenous nation affiliation and/or other markers or proxies for Indigenous affiliation, such as ethnic identifiers. We strongly urge reconsidering the use of race as a proxy for Indigenous affiliation or identification. Racialized data for Indigenous Peoples often assumes homogeneity of an Indigenous community which may lead to damaging and essentialized genetic conclusions and adopts settler-colonial tools of race-making and Indigenous erasure (Russel, 2005; Tallbear, 2013; Ratteree and Hill, 2017; Rodriguez-Lonebear, 2020). Indigenous affiliation and other identifiers more appropriately represent the rights of Indigenous Peoples to define who belongs to their communities.

During the time of COVID-19, the need for disaggregated data on Indigenous Peoples at various geographic levels is needed. Without this, COVID-19 cases and death rates are obscured due to their small population sizes relative to the majority population. As a result, it is difficult to identify emerging hotspots for infections and the need for taking preventative actions.

The United Nations, Department of Economic and Social Affairs (2020); World Health Organization (2020), and the Pan American Health Organization (del Pino and Camacho, 2020) call for the inclusion of identifiers or affiliation for Indigenous Peoples and individuals in COVID-19 data (direct and indirect) and the need to make those data available to decision-makers and Indigenous Peoples. In the US, there has been a call for the availability of data disaggregated by Indigenous nations and tribal affiliation themselves, with the caveat that this must be done within the context of Indigenous governance of those data, including data sharing agreements (Urban Indian Health Institute, 2020).

### Increasing the Number of Indigenous Epidemiologists to Improve Information for Effective Public Health Response

Realizing Indigenous Peoples capability in responding to health emergencies requires increasing the number of Indigenous epidemiologists practicing in Indigenous communities and at other institutions. Dedicated training and education pathways for Indigenous people are needed to bolster public health expertise and to decolonize public health practice. This is in recognition that epidemiology and public health practice has a long history of harm under the guise of protecting public health (Parsons, 2008; Prussing, 2019; Cormack and Paine, 2020). It also aims to negate the influx of outsiders who are required to be brought up to speed or think that generic cultural awareness makes for safe practice (Kurtz et al., 2018).

An Australian program could serve as a model, the field-based epidemiology training with Aboriginal and/or Torres Strait Islander people based on the Centres for Disease Control Field Epidemiology Training Program results in 50% of trainees going on to PhDs and working in public health, environmental health and academia (Guthrie et al., 2011).

## Conclusion

Indigenous Peoples suffer inequitable direct and indirect effects of the COVID-19 pandemic. A number of Indigenous Peoples, international organizations, journalists, and scholars have shed light on COVID-19 challenges and successes for Indigenous Peoples. Many have provided recommendations for the advancement of Indigenous rights and interests during the pandemic. However, with respect to data, few have gone beyond calling for disaggregated Indigenous COVID-19 related data. Since data are critical for decision-making for pandemic planning, mitigation, and response, and Indigenous Peoples' participation in data stewardship can increase the benefits of data use and decrease the harms, the involvement of Indigenous Peoples' with COVID-19 data is of paramount importance. The recommendations outlined here serve to increase Indigenous Peoples governance of and access to data across data lifecycles and data ecosystems for an enhanced response to the COVID-19 pandemic. Implementation of these recommendation can lead to better pandemic responses and planning for future events for Indigenous Peoples worldwide. In a global context where diseases know no boundaries, improvements in response in Indigenous communities protects everyone by closing all lingering reservoirs and refugia for the virus to propagate, mutate, or re-initiate after a seeming cessation of cases. Addressing the effects of COVID-19 on Indigenous Peoples necessitates enhancing Indigenous nations and communities' data infrastructures and access.

## Author Contributions

SC conceptualized, drafted, edited, and coordinated the manuscript. RA, PC, DC, TK, RL, MS, and RR contributed to the overall conceptualization, writing, and editing. All authors contributed to the article and approved the submitted version.

## Conflict of Interest

The authors declare that the research was conducted in the absence of any commercial or financial relationships that could be construed as a potential conflict of interest.

## Footnotes

^1^https://data.humdata.org.
